# It's in the mix: psychological distress differs between combinations of alexithymic facets

**DOI:** 10.3389/fpsyg.2014.01259

**Published:** 2014-11-12

**Authors:** Elif Alkan Härtwig, Claudia Crayen, Isabella Heuser, Michael Eid

**Affiliations:** ^1^Cluster of Excellence “Languages of Emotion,” Freie Universität BerlinBerlin, Germany; ^2^Department of Psychiatry, Charitè University MedicineBerlin, Germany; ^3^Department of Education and Psychology, Freie Universität BerlinBerlin, Germany

**Keywords:** alexithymia, psychological distress, latent profile analysis, subtypes, TAS-20, BVAQ, SCL-90-R

## Abstract

Alexithymia is a personality trait characterized by difficulties in identifying, describing, and communicating one's emotions. The aim of the present study is to examine the usefulness of a typological approach considering the interaction between distinct alexithymic features within a population of high-alexithymic German adults (*N* = 217). Latent profile analysis (LPA) was employed to test for possible underlying profiles. A 3-profile solution showed the best fit*:* The profiles can be described as (1) “low”: lower load on all facets of alexithymia, (2) “mixed”: specific problems on identifying emotions, and (3) “high”: higher load on all facets of alexithymia. Moreover, this study tested how these profiles differed in psychological distress. “Mixed” profile, with specific problems on identifying emotions showed the highest levels of psychological distress. The present study suggests the importance of a specific combination of alexithymic features, rather than total alexithymia scores, as a risk factor for psychological distress.

## Introduction

Alexithymia is a personality trait that is associated with some psychiatric disorders (Lumley et al., 1996; Taylor and Bagby, 2004) such as eating disorders (Nowakowski et al., 2013), pain syndromes (Huber et al., 2009), and somatoform disorders (De Gucht and Heiser, 2003; Mattila et al., 2009). In turn, patients with psychiatric disorders often show state-dependent alexithymic features (Honkalampi et al., 2000, 2010; Leweke et al., 2012). Sifneos (1973) introduced the term alexithymia in an attempt to explain his observations on “psychosomatic” patients, who had deficits in verbal expression of emotions. The original alexithymia construct included four components: (a) difficulty identifying subjective feelings, i.e., the inability to differentiate between different feelings and to distinguish feelings from bodily sensations of emotional arousal; (b) difficulty describing feelings, i.e., a compromised capacity to use language to symbolize emotions; (c) a limited imaginative capacity and fantasy life; (d) a utilitarian way of thinking with a stimulus bound, externally oriented cognitive style (Nemiah et al., 1976; Taylor et al., 2003).

The most frequently used psychometric instrument for alexithymia is the self-report 20-item Toronto Alexithymia Scale (Bagby et al., 1994a). It consists of three components of the original alexithymia construct of Sifneos (1973) only: Difficulty identifying feelings (DIF), difficulty describing feelings (DDF), and an externally oriented thinking (EOT) style. The original facet of a restricted fantasy life has not been included in TAS-20. Parker et al. (2003) later on demonstrated that it is part of the EOT subscale (Bagby et al., 1994b).

Vorst and Bermond (2001) constructed a questionnaire based on a broader operationalization of alexithymia. The Bermond-Vorst Alexithymia Questionnaire (BVAQ; Vorst and Bermond, 2001) consists of five subscales, one of which is *Emotionalizing*, i.e., the extent to which someone is emotionally aroused by emotion inducing events. They also included *Fantasizing* explicitly into their questionnaire.

In empirical studies the multidimensional structure of alexithymia is usually measured by correlating the different subscales of an alexithymia questionnaire with other variables. This common approach ignores that there might be strong interactions between the different facets of alexithymia and that different combinations of facet scores might contain more information than considering the facets separately. The interactions between different facets can be quantified in a multiple regression analysis by including different interaction terms. This way of handling interaction, however, has two shortcomings. The first shortcoming is that many interaction terms have to be considered. With respect to the five subscales of the BVAQ, for example, 10 two-way interaction terms, 10 three-way interaction terms, five four-way interaction terms, and one five-way interaction term must be included. It is likely that adding these twenty-six additional independent variables into an analysis can lead to unstable results that are difficult to communicate. The second shortcoming is that this approach can only be used when the interactions are considered on the level of alexithymia as an independent variable, but not if one is interested in alexithymia as a dependent variable. In order to overcome these shortcomings a typological psychometric approach can be used. There are few studies using a typological approach. For example Moormann et al. (2008) proposed the idea of alexithymia subtypes. They created these subtypes based on factor scores (high vs. low) on two higher order-factors that underlie the BVAQ: A cognitive factor (*Identifying, Verbalizing*), and an affective factor (*Emotionalizing, Fantasizing*), with *Analyzing* loading on both factors. However, the study of Bagby et al. (2009), using CFA and cluster analysis in a large sample, did not support the two higher-order factor structure of the BVAQ and the subtypes proposed by Moormann et al. (2008). From a psychometric point of view the factor score approach used in these studies has some weaknesses. Factor analysis provides information about the grouping of questionnaire items but not about where to draw the line between individuals with “high” and “low” factor scores, so that the grouping is arbitrary. Also, unlike the approach used here, cluster analysis does not explain the dependencies between observed variables and does not provide a clear rationale for choosing the appropriate number of clusters. From a psychometric point of view, latent profile analysis (LPA) is more appropriate. To our knowledge, no study to date has used LPA for a refinement of the discussion on alexithymia subtypes. This research aims to detect different latent subgroups (profiles) of alexithymia, and to explore whether these subgroups differ in psychological distress and other variables such as personality traits and measures of emotional experience.

LPA is an appropriate typological model when continuous observed variables are considered (Lazarsfeld and Henry, 1968). LPA assumes that the population consists of several subpopulations (latent classes) that are distinct (non-overlapping) and exhaustive (all latent classes together build the population). The latent classes differ in the profile of the mean values of the observed variables. Within the latent classes the observed variables are independent. This implies that the latent classes explain the dependencies between the observed variables. However, LPA can be extended to allow dependencies within latent classes that also can be represented by a multidimensional model (e.g., mixture distribution factor models). LPA belongs to the family of mixture distribution models. Special cases within this family are purely multidimensional models and purely typological models. In latent profile analyses, a latent categorical variable is defined which can be used as a dependent as well as an independent variable. Moreover, subjects can be assigned to latent classes based on their assignment probabilities. The mean assignment probabilities for each latent class indicate the reliabilities of the assignments. Hence, LPA can also be used in psychological assessment. Because of these advantages LPA is a useful tool for considering complex interactions of the facets of a multidimensional trait. The major aim of the present study is to explore the usefulness of LPA for alexithymia research. Moreover, we sought to analyze whether the different alexithymia profiles differ in other psychological variables such as personality traits, emotional experience and, in particular, psychological distress.

### Alexithymia and psychological distress

Alexithymia embodies several dysfunctionalities in emotion processing. It is a similar concept to emotional intelligence (EI; Salovey and Mayer, 1989/1990), which covers on the one hand the ability to access one's own emotional states, on the other hand the ability to understand the emotional states of others. Alexithymia, capturing essentially the first, is similar to and overlapping with EI but they are still two independent constructs (Parker et al., 2001). Higher levels of EI are related to sound mental health (Lizeretti et al., 2012). The opposite applies for alexithymia, where the higher scores are linked to many psychiatric disorders.

Already in seventies Sifneos (1973) argued that alexithymia predisposes individuals to “classical psychosomatic” disorders. Recent studies showed that psychiatric patients tend to score high on alexithymia scales (Porcelli et al., 2004; Frewen et al., 2008; Leweke et al., 2012; De Berardis et al., 2013; Robinson and Freeston, 2014). Although these studies point to a relationship between alexithymia and psychiatric disorders, they do not provide information on the extent to which alexithymia predisposes individuals to psychiatric disorders in normal population. Recently Liang and West (2011) and Leising et al. (2009) provided evidence that alexithymia is highly correlated with psychological distress in non-clinical populations. Mattila et al. (2009) demonstrated in a large population based survey that alexithymia is negatively linked to health related quality of life. Especially the scale DIF found to be strongly related to psychiatric and somatic symptoms. Hence it is important to understand whether alexithymia in itself is a risk factor for psychological distress or the interaction between different facets of alexithymia makes a person more or less susceptible to psychological distress.

Therefore, we will use LPA to explore if certain profiles of alexithymia facets are linked more closely to psychological distress than others. Liang and West (2011) demonstrated that difficulty identifying and describing feelings correlates with psychological distress, while EOT style does not. However, they didn't consider the interactions between different alexithymia facets. In the present study, similar to research before (Leising et al., 2009; Liang and West, 2011), the frequency of reported psychiatric symptoms on the well-established SCL-90-R scale will be used as a marker of psychological distress.

## Methods

### Measures

#### Measures of alexithymia

***TAS-20***, The 20-Item Toronto Alexithymia Scale is a self-report instrument with replicated validity and reliability (Bagby et al., 1994a,b; Taylor et al., 2003). It gives a total score that is derived from three subscales; DIF (seven items such as: “*I am often puzzled by sensations in my body*.”), Difficulty Describing Feelings (DDF; five items such as: *“It is difficult for me to find the right words for my feelings.”*), and External Oriented Thinking (EOT; eight items such as: *“I prefer to just let things happen rather than to understand why they turned out that way.”*). The items are rated on a Likert Scale ranging from 1 to 5, resulting in a maximum score of 100 points. Higher scores indicate a higher load of alexithymia. The factorial structure of TAS-20 has been demonstrated being stable and valid in the English version (Parker et al., 2003) as well as in other European and non-European languages (Taylor et al., 2003). The first two factors (DIF and DDF) of the German version have satisfactory internal consistencies (Cronbach's as from 0.69 to 0.81; Bach et al., 1996). It has been repeatedly found that the third factor (EOT) has lower a scores (0.55 to 0.61), despite the fact that it is the scale with the highest number of items (Bach et al., 1996; Parker et al., 2003). The average inter-item correlations of the whole TAS-20 and each subscale are also acceptable (0.23 for TAS-20, 0.37, 0.40, 0.24 for the subscales DIF, DDF, EOT, respectively) (Parker et al., 2003).

***BVAQ***. The Bermond-Vorst-Alexithymia-Questionnaire (Vorst and Bermond, 2001) consists of five subscales, each scale comprising eight items. It was developed in Dutch but has been validated in many other languages, including German (Müller et al., 2004). The five subscales of the BVAQ are:

1. Difficulty in *Emotionalizing*: The degree of emotionally arousal by emotion inducing events (e.g., *“When I see somebody crying uncontrollably, I remain unmoved.”*).
2. Difficulty in *Fantasizing*: The degree to which someone tends to fantasize, imagine, day-dream (e.g., “*Before I fall asleep, I imagine all kinds of events, encounters, and conversations*.”).
3. *Identifying*: Difficulty in defining one's own arousal states (e.g., “*When I am tense, it remains unclear from which of my feelings this comes*.”).
4. *Analyzing*: The restrained tendency to seek explanation for one's own emotional reactions, (e.g., “*When I feel uncomfortable, I will not trouble myself even more by asking myself why*.”).
5. *Verbalizing*: The extent to which someone is able to communicate one's own emotional states and reactions (e.g., *“I find it strange that others analyze their emotions so often.”*) (Vorst and Bermond, 2001).

Each item is rated on a 1 to 5 point Likert scale. The maximum possible score is 200 with higher scores indicating higher alexithymia. The total BVAQ score has a Cronbach's a coefficient of 0.83, which is highly satisfactory although the a scores of the subscales range from 0.54 to 0.80, with *Emotionalizing* having the lowest internal consistency (Müller et al., 2004).

#### Measure of personality dimensions

***NEO-Five Factor Inventory (NEO-FFI)***. This inventory consists of 60 self-report items rated on a 0 to 4 point Likert scale with higher scores indicating a higher level of that personality dimension: Neuroticism (N), Extraversion (E), Openness (O), Agreeableness (A), and Conscientiousness (C). The German translation by Borkenau and Ostendorf (1993) is used in the present study. The NEO-FFI is a condensed form of the NEO-Personality Inventory (Costa and McCrae, 1992) and has an adequate internal consistency (a of each scale ranging from 0.64 to 0.80; Müller et al., 2004), temporal stability, and construct validity.

#### Measure of emotional experience

***The scale for attention to feelings and clarity of feelings***. The scale for the assessment of attention to feelings and clarity of feelings (Lischetzke et al., 2001) consists of 12 items that are rated on a 1 to 4 point Likert scale. The instrument was developed in German and has strong psychometric properties with a stable two-factor structure and high internal consistencies of 0.87 for attention to and 0.88 for clarity of feelings (Lischetzke et al., 2001).

#### Measure of psychological distress

***Symptom Check List-90-Revised***. The SCL-90-R (Derogatis, 1977; Franke, 1995) is widely used internationally for the assessment of psychological distress. It consists of 90 items with nine subscales and has demonstrated adequate reliability and satisfactory construct validity. The PST (*Positive Symptom Total*, absolute number of exhibited symptoms) is used in the analysis as an indicator for general psychological distress.

### Sample

Subjects were recruited via an announcement in the public transport system. They completed an online version of the TAS-20. Those who had a score higher than 56 were invited for further investigation. Thus, the sample included highly alexithymic individuals (HA) only. The present analyses focus on differences within this particular HA group that were commonly overlooked in the past. Having high scores on alexithymia has a demonstrable relationship with psychiatric disorders. Therefore, the current sample only includes individuals with very high scores on alexithymia, in order to gain insight on the degree of psychological distress among possible distinct latent profiles.

Since alexithymia is a stable trait only after late adolescence (Mattila et al., 2006), subjects younger than 22 years of age were excluded from the analysis. Mean age of the sample was 35.5 (*SD* = 11.4). The sample included 96 women (44.2%), and 98.3% of all participants had at least a secondary school degree. The final sample consisted of 217 individuals who all reported German to be their first language. Participants received an information leaflet and €30 each for their participation. The study was compatible to the requirements of the Helsinki Agreement and approved by the institutional ethics committee.

### Procedure

Participants were invited to attend a group session to complete the research questionnaires. After reading and signing the consent form, subjects filled out the questionnaires on a personal computer.

In the group session, the BVAQ, the NEO-FFI, the scales for attention to feelings and clarity of feelings and the SCL-90-R were administered. The means and standard deviations of all instruments can be found in Table 1 (see Supplementary Material for the correlations among all studied measures).

**Table 1:** **Means and SDs of the applied instruments for the total sample (*N* = 217)**.

	**No. of items**	**Range**	**Mean**	***SD***
TAS-20 total	20	56–96	68.2	7.0
Difficulty identifying feelings	7	10–35	24.4	4.3
Difficulty describing feelings	5	14–25	20.4	2.6
Externally oriented thinking	8	14–38	23.3	4.3
BVAQ Total	40	91–174	131.3	15.9
Verbalizing	8	21–40	33.5	4.4
Identifying	8	16–40	28.9	5.1
Analyzing	8	9–39	22	6.7
Fantasizing	8	8–38	21.5	7.4
Emotionalizing	8	17–35	25.3	3.5
**NEO**–**FFI**				
Openness	12	0.5–3.5	2.5	0.6
Neuroticism	12	0.1–4.0	2.3	0.8
Extraversion	12	0.5–3.2	1.8	0.6
Agreeableness	12	0.2–3.6	2.3	0.6
Conscientiousness	12	0.6–4.0	2.5	0.7
Attention to feelings		1.0–4.0	2.2	0.7
Clarity of feelings		1.0–3.8	1.8	0.5
**SCL-90-R**				
PST		0–81	41.2	16.9

*TAS-20, Toronto Alexithymia Scale (with a 1–5 point Likert Scale, 100 is the maximum total score); BVAQ, Bermond-Vorst Alexithymia Questionnaire; NEO-FFI, NEO-Five Factor Inventory; SCL-90-R, Symptom Check List-90-Revised; PST, Positive Symptoms Total*.

### Statistical analyses

All analyses were conducted using the software Mplus (Version 7; Muthén and Muthén, 1998/2012). First, a LPA (LPA; Lazarsfeld and Henry, 1968) was conducted and distinct mean score profiles within the high alexithymic (HA) sample were identified. LPA is distinct from latent class analysis in the traditional sense because of its use of continuous indicators. Common to both mixture approaches is the assumption of population heterogeneity or qualitatively different subtypes underlying the observed data structure. In LPA, subtypes are characterized by their distinct profile of mean scores on the indicator variables.

Second, following the procedure suggested by Vermunt (2010) and implemented in Mplus 7 (Asparouhov and Muthén, 2012), sets of external variables were related as covariates to the identified latent profiles in a modified three-step method. This method avoids two problems commonly associated with the prediction of latent classes. On the one hand, the measurement model that is used to decide on the number of mixture components is not influenced by the covariates as it would be in a simultaneous estimation. This has particular importance in studies such as the present one, where the number of covariates is large and the relationships between covariates and profiles are investigated in a more exploratory manner. On the other hand, the common approach to assign each individual to his or her most likely profile and treating profile membership as a manifest variable in a multinomial logistic regression model reintroduces classification error. As a result, regression coefficients would be biased. In contrast, the modified three-step method links the assigned profile membership to the latent profile by using the classification error probabilities as weights. The estimates for the effects of covariates on these reconstructed latent profiles are minimally biased. For all analyses the significance level is α = 0.05.

#### Latent profile analysis

The aim of LPA is to determine the number and character of unobserved subtypes to account for the mean and covariance structure found in the dataset. In this study, indicators for the LPA were the individual sum scores of the five BVAQ subscales; *Identifying, Verbalizing, Analyzing, Fantasizing*, and *Emotionalizing*. The TAS-20 subscales were treated as external variables instead of indicators, because they had already been used in the process of sample selection and had been assessed in a non-laboratory (online) setting. As a baseline, the model served as a saturated model that perfectly reproduced the sample's mean and covariance structure. Different LPA models, with the number of profiles ranging from two to five were then compared to the baseline model and to each other. The assumption of conditional independence was kept; none of the subscales were allowed to correlate within each latent profile. All models were estimated using maximum likelihood with robust standard errors. The Mplus syntax for the model setup is available from the second author.

There is still some disagreement concerning the rules in determining the number of groups in LPA models (e.g., Lubke and Muthén, 2005; Marsh et al., 2009). One possibility is to compare models by information criteria (Read and Cressie, 1988). These statistics take model parsimony into account, so that smaller index values indicate a better fit in relation to model complexity. There is evidence that the Bayesian Information Criterion (BIC) performs best in mixture models (Nylund et al., 2007). We thus gave the BIC priority in interpreting our results. We also include the results of the Vuong-Lo-Mendell-Rubin likelihood ratio test (VLMR-LRT; Lo et al., 2001) that tests the null hypothesis that a model having one class less than the model considered has generated the data. A significant result means that the model considered should be favored over a model with one class less.

#### Logistic regression analyses

In order to get a more detailed picture of the character of the obtained profiles, multinomial logistic regression models were specified to predict profile membership in the context of the modified three-step-method. Three sets of predictors were specified and tested in separate models: (A) the TAS-20 subscales and the number of positive symptoms (PST of SCL-90-R) as an indicator of psychological distress; (B) the five NEO-FFI personality dimensions, and (C) attention to feelings and clarity of feelings.

## Results

### Latent profile analysis

Information criteria and additional goodness-of-fit measures for the baseline model and the LPA models can be found in Table 2. According to BIC, the 3-profile-model should be considered. Further support for the 3-profile-model comes from the VLMR-LRT. The small *p*-value < 0.05 for the 3-profile-model indicates that a 2-profile model must be rejected. The larger *p*-value for the 4-profile-model indicates that a 3-profile model can safely be utilized. In addition, all three profiles in the 3-profile solution have substantial sizes and high mean assignment probabilities (Profile 1: 0.90; Profile 2: 0.87; Profile 3: 0.88), which demonstrate that the reliability of assigning individuals to the profiles is high.

**Table 2:** **Goodness-of-fit measures for different numbers of profiles**.

	**Baseline model**	**No. of extracted profiles**			
		**2**	**3**	**4**	**5**
BIC	6692	6691	6681	6692	6705
*p*(VLMR-LRT)		0.0003	0.0456	0.1318	0.1394
Smallest group (%)	100	28	25	8	7

*BIC, Bayesian Information Criterion; VLMR-LRT, Vuong-Lo-Mendell-Rubin Likelihood-Ratio-Test*.

**Figure 1:**
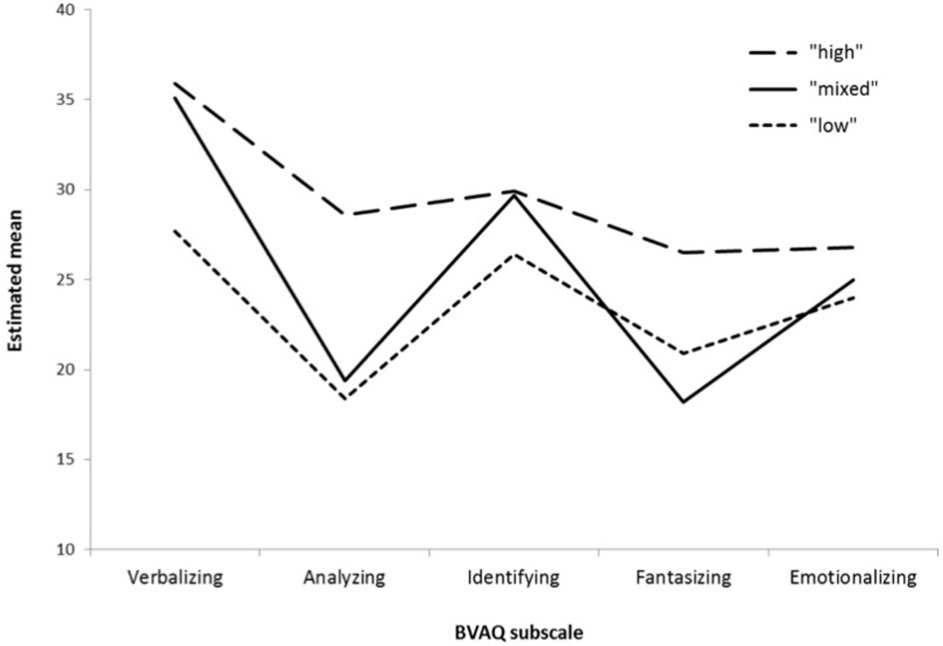
**Estimated means of the 3-profile solution**. Possible score range on BVAQ subscales is 8-40.

The patterns of the estimated means on the BVAQ subscales for the three profiles are depicted in Figure 1. High mean levels imply high difficulties in the corresponding domain. The different profiles can be characterized as follows: One profile is distinguished by high mean levels on all subscales and comprises about 32% of the sample (dashed line in Figure 1). Within the population of HA, the individuals belonging to this profile report high levels of difficulty in all domains represented by the BVAQ subscales. This will be referred to as the “high” profile. In contrast, there's another profile that consists of 25% of the sample and exhibits considerably lower mean levels on all BVAQ subscales (dotted line in Figure 1), which we will refer as “low” profile. Besides “low” and “high” profiles, there is a third profile that comprises the remaining 43% of the sample (solid line in Figure 1). This profile does not simply show intermediate mean score levels, but reveals a distinct pattern with profound differences depending on the subscales: On the subscales *Verbalizing* and *Identifying*, it matches the level of the “high” profile. On the subscales *Analyzing* and *Fantasizing*, it matches the level of the “low” profile. The mean for *Emotionalizing* lies in between the other two profiles. This will be referred to as the “mixed” profile. It represents a particularly interesting subgroup of HA individuals, because the corresponding participants report severe difficulties in identifying and describing feelings, that are typical for alexithymia, but do not feel equally restricted in their fantasy life or describe their thinking style to be strongly externally oriented. In the next section, different sets of external variables are linked to the profiles in order to explore how these aspects of alexithymia are embedded and intertwined with broader constructs of personality, emotional experience and psychological distress.

### Logistic regression

In this section, we will relate certain non-alexithymia measures to the identified profiles and see which characteristics allow us to predict whether someone is assigned to one particular profile rather than another. In choosing the “mixed” profile as reference group in the multinomial logistic regression, we put emphasis on contrasting the “mixed” profile with the general “low” and general “high” profiles. The “high” and “low” profiles are already distinguishable by their BVAQ mean scores and related measures. The regression coefficients for the three predictor-sets in separate multinomial logistic regression models are shown in Table 3. Note that all coefficients are in logit form and interpreted as partial regression coefficients, adjusted for all other effects in the same model.

**Table 3:** **Logistic regression coefficients for predictor sets A to C**.

**Set**	**Scale**	**Non-reference profiles**							
		**“High”**				**“Low”**			
		***B***	***SE***	***|z|***	***p***	***B***	***SE***	***|z|***	***p***
A	TAS-DIF	−0.09	0.09	1.01	0.311	0.18	0.15	1.18	0.237
	TAS-DDF	−0.12	0.16	0.79	0.432	−0.81	0.19	4.20	<0.001
	TAS-EOT	0.30	0.09	3.41	0.001	−0.10	0.11	0.93	0.352
	Positive symptoms	−0.05	0.02	2.67	0.008	−0.06	0.03	2.45	0.014
B	Neuroticism	−1.82	0.81	2.27	0.024	−0.33	0.39	0.85	0.393
	Extraversion	−0.49	0.61	0.81	0.417	0.88	0.56	1.57	0.116
	Openness	−4.03	1.59	2.53	0.011	−0.69	0.72	0.96	0.335
	Agreeableness	0.01	0.61	0.01	0.994	1.05	0.58	1.80	0.071
	Conscientiousness	0.20	0.52	0.39	0.696	0.47	0.44	1.07	0.283
C	Clarity of feelings	−0.68	0.70	0.98	0.327	2.70	0.73	3.70	<0.001
	Attention to feelings	−5.42	1.13	4.80	<0.001	0.38	0.57	0.67	0.503

*The “mixed” profile serves as reference group. |z| is the absolute value of the ratio B/SE. B, the coefficient on the independent variable; SE, Standard Error*.

#### TAS-20 subscales and the number of positive symptoms

As the EOT scores increase, the probability of being assigned to the “high” profile rather than the “mixed” profile increases. This is not surprising, given the strong overlap of the TAS-20 EOT scale with the BVAQ *Analyzing* scale in which these two profiles differ. Similarly, to be assigned to the “low” profile compared to the “mixed” profile becomes less likely with higher DDF (difficulties in describing feelings) scores. DIF has no significant effect, which corresponds to Figure 1, which shows the differences between the profiles on the *Identifying* scale are not as pronounced. The fourth predictor in this model, number of positive symptoms on the SCL-90-R has an interesting effect. When number of reported symptoms increase, it becomes less likely to be assigned to either the “high” or the “low” profile. To illustrate the magnitude of this effect, imagine a HA individual with an average number of positive symptoms (41, see Table 1) compared to an extremely distressed HA individual with the maximum reported number of 81 symptoms. Let both have equal scores on the TAS subscales. The chances for the extremely distressed HA individual to be assigned to the “mixed” profile rather than to the “high” profile are about seven times higher than for the moderately distressed HA individual [*B*_pst_ = −0.05; OR = *e*^(*B*pst^) = 0.95; Difference of 40 symptoms gives 0.95^40^ = 0.14; Inverse for “mixed” compared to “high” is 1/0.14 = 7.14]. In short, the subjects assigned to the “mixed” profile exhibit more psychological distress than the individuals assigned to the other two profiles. We will elaborate on this point in the discussion.

**Figure 2:**
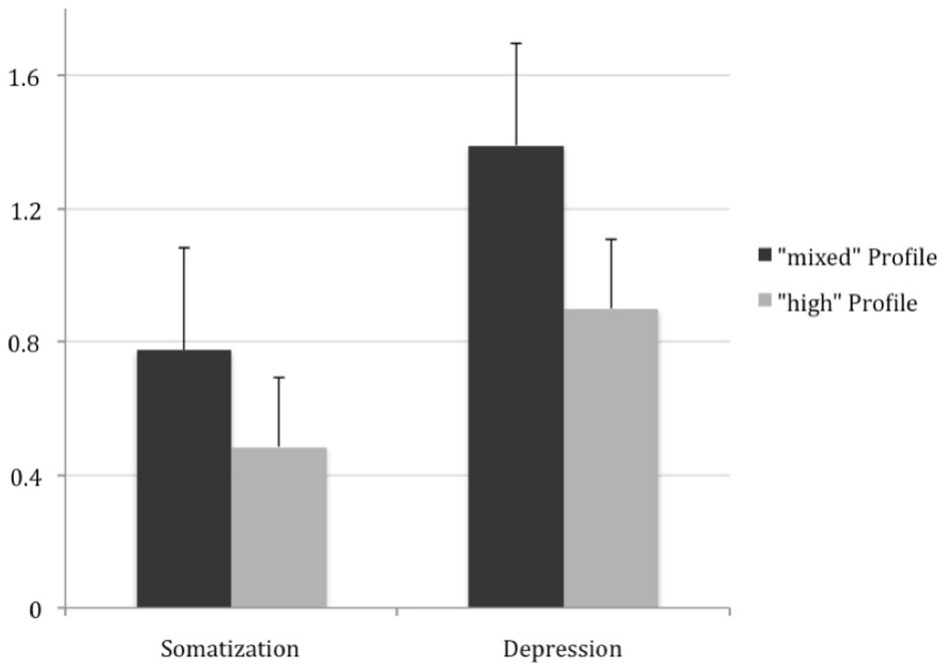
**Scores from Somatization and Depression subscales of SCL-90-R**. High-distressed individuals have significantly higher number of somatization [*t*_(102)_ = 2.3317; *p* = 0.0217] and depressive symptoms [*t*_(102)_ = 2.8812; *p* = 0.0048].

Furthermore, as a complementary analysis, the “mixed” and “high” profiles were compared based on the depressive and somatization symptoms of SCL-90-R. The members of the “mixed” profile have significantly higher scores on Somatization and Depression Subscales of SCL-90-R (See Figure 2 for details).

#### Personality dimensions

Of all personality dimensions (NEO-FFI) only neuroticism and openness have a significant effect. The negative logits for neuroticism and openness indicate that higher levels of these dimensions have an increasing probability of being assigned to the “mixed” profile compared to the other two profiles.

#### Attention to feelings and clarity of feelings

This model reveals an interesting pattern of effects. There are differential effects for the “high” and “low” profiles. The “high” profile is distinguishable from the “mixed” profile by a strongly decreased level of attention to feelings, whereas the “low” profile is distinguishable from the “mixed” profile by an increased, but not significantly different level of clarity of feelings. The combination of high attention to feelings and low clarity of feelings strikes us as the peculiarity of the “mixed” profile.

In summary, the “mixed” profile is characterized by higher psychological distress, neuroticism, and openness compared to the remaining two profiles. It can be distinguished from the “high” profile by a higher level of attention to feelings and from the “low” profile by lower clarity of feelings.

## Discussion

The goal of this study was to explore a specific combination of alexithymic features as a risk factor for psychological distress by means of LPA. LPA revealed a 3-profile solution. Each independent profile was characterized by a distinct mean score pattern on alexithymia subscales as well as on external measures of personality (NEO-FFI), emotional processing (attention, clarity), and psychological distress (PST).

Contrary to general expectations, this study revealed that individuals with the highest alexithymia scores had the lowest levels of psychological distress. However, higher levels of alexithymia have been repeatedly found related to many psychiatric disorders (Taylor and Bagby, 2004). The present study highlights the importance of considering the scores on the alexithymia subscales and their specific combinations rather than merely using the total score. The combination of distinct difficulties in identifying and describing feelings paired with a rather low tendency to EOT plays a role in the relation commonly found between alexithymia and psychological distress.

Two other dimensions that appear to be useful in distinguishing this “mixed” group from “high” and “low” alexithymic individuals are attention to and clarity of feelings. Whereas attention and clarity are both relatively high in the “low” group, and the “high” group exhibits low attention to and clarity of feelings, the “mixed” group is characterized by high attention and low clarity at the same time. In the following, we will discuss why this “mixed” group is more prone to psychological distress than the “high” group with the highest total scores. The present study suggests a mismatch between attention to one's own feelings and the clarity of feelings in the “mixed” group. These individuals reported paying attention to their feelings but were still unclear about them. This discrepancy between the need to understand and the inability in understanding one's feelings leads to frustrations and likely explain why the “mixed” group has a higher tendency toward psychological distress. However, “high” profile individuals pay less attention to their unclear feelings, so that the proposed mismatch is absent in this group. These results are comparable with the findings of Lizeretti and Extremera (2011) and Lizeretti et al. (2012) which found higher attention to feelings in clinical subjects compared to non-clinical control subjects. Attention to and clarity of feelings are two very important domains of EI (Salovey et al., 1995). The demonstrated mismatch, between attention to and clarity of feelings in the “mixed” profile of alexithymia with highest levels of psychological distress, highlights the similar effects of alexithymia and EI on psychological distress.

The facet of alexithymia in which “mixed” and “high” profile individuals manifested a difference is *Fantasizing*. The “high” group had a distinct impairment in fantasizing. On the contrary, the “mixed” individuals reported fewer difficulties in fantasizing, and they showed higher values on openness to experiences (NEO-FFI). Impaired ability to fantasize is one of the main facets of the original alexithymia construct (Nemiah et al., 1976). Bagby et al. (1994a) did not include this dimension in the TAS-20 because of its relation to social desirability (Parker et al., 2003), and they argue that EOT already measures fantasizing, albeit indirectly (Bagby et al., 1994b). Although not being included in their instrument, the authors of the TAS-20, however, argue similar to Vorst and Bermond (2001) that fantasizing belongs to the core concept of alexithymia. Further research is needed to get a deeper understanding of the effects of *Fantasizing* on psychological well-being.

Aside from *Fantasizing* the main difference between “mixed” and “high” profile individuals lies in the difficulty in *Analyzing* (understanding and decoding) one's own emotions and the EOT style. EOT is one of the main facets of the original alexithymia definition (Nemiah and Sifneos, 1970) and part of both self-report instruments. It represents the concept of pensée opératoire (Marty and M'Uzan, 1963), which is defined by the tendency of having a cold, technically oriented thinking style. In our sample, the typical alexithymic individuals with high scores on all dimensions including *Analyzing* experience significantly less psychological distress than the “mixed” profile individuals, who score particularly low in *Analyzing* and *Fantasizing*. Although research mainly focused on difficulties in identifying and describing of feelings as core domains of alexithymia, our research suggests high EOT and low *Fantasizing*, might actually have a protective value for alexithymic individuals.

Our findings indicate a possible inaccuracy of the commonly drawn direct link between alexithymia and psychiatric symptoms. The notion that high scores of alexithymia in itself may lead to psychosomatic disorders because of the failure in interpreting physical arousal that accompanies emotional experiences (Taylor et al., 1991; De Gucht and Heiser, 2003; Taylor and Bagby, 2004; Mattila et al., 2008) especially should be examined in future studies. Alexithymia is conceived as a multidimensional construct and is measured as such by the TAS-20 and the BVAQ. It is only consistent to test hypotheses regarding risk factors for psychiatric diagnoses on the level of the distinct subscales. In addition to general scores of psychological distress, it is important to point out that “mixed” profile individuals have much higher somatization and depressive symptom-frequency than the “high” profile individuals, supporting the earlier findings that DIF is especially related to somatization (Kooiman et al., 2000; Waller and Scheidt, 2006). It is necessary to consider the role of different alexithymia facets and the interplay between those facets and that LPA is an appropriate psychometric model for detecting such types.

The present study employed a sample of high-alexithymic individuals. Since alexithymia is a dimensional, not a categorical construct, one might argue against the use of a group of individuals over a specific cut-off score. This method is used because of the acknowledged risk of high-alexithymic individuals developing psychiatric disorders. Therefore, we specifically sampled from the far right end of the normal distribution of alexithymia in order to enrich the sample accordingly. We are aware that there might be other latent profiles underlying the total spectrum. Yet the focus was on the differentiation within high-alexithymic individuals. An unfiltered sample has the potential to cloud these specific differences within high-alexithymic individuals, since the latent profiles extracted would be dominated by profiles differentiating between the more common medium levels of alexithymia facets. Further research is needed to explore the latent profiles underlying the whole spectrum of alexithymia.

Positive aspects of the current study are the use of a large community based sample with a wide age range and the application of both of the commonly used questionnaires for alexithymia. A limitation of the study is that the study depended solely on self-report instruments. Because of the impairments of alexithymic individuals in the domains of emotional self-awareness and verbal expression, using this method for determining the levels of alexithymia might be problematic (Stingl et al., 2008; Meganck et al., 2009). More research is necessary to explore the alexithymia construct as a risk factor for psychiatric disorders using objective measures of alexithymia, such as the Toronto Alexithymia Interview (Bagby et al., 2006), and the Observer Alexithymia Scale (Haviland et al., 2000). Furthermore, it is not clear if the HA population has the introspective capacities to make accurate judgments on their personal distress. Although SCL-90-R is a widely used instrument with clear and simple descriptions of psychiatric symptoms, a joint assessment based on the judgment of an experienced clinician and self-report instrument would increase the reliability of the results.

In conclusion, our results highlight the heterogeneity of the alexithymia construct. Dynamics between subscales of alexithymia are shown to be crucial determinants for psychological distress rather than merely the total alexithymia score. Our study reveals that the commonly held notion of high alexithymia score being a determining risk factor for psychological health should be reconsidered.

### Conflict of interest statement

The authors declare that the research was conducted in the absence of any commercial or financial relationships that could be construed as a potential conflict of interest.
