# Supplementary material for: It's in the mix: psychological distress differs between combinations of alexithymic facets
Source: Front Psychol. 2014 Nov 12;5:1259. doi: 10.3389/fpsyg.2014.01259 (PMC4228974; doi:10.3389/fpsyg.2014.01259)
Supplement: Supplementary file 1 [file Appendix.PDF]

Appendix

Correlations among all studied measures

	TAS-20	DIF	DDF	EOT	BVAQ	Verbalizing	Fantasizing	Identifying	Emotionalizing	Analyzing	Neuroticism	Extraversion	Openness	Agreeableness	Conscientiousness	PST	Clarity of feelings	Attention to feelings
TAS-20	1	**.65****	**.54****	**.62****	**.42****	**.35****	,04	**.29****	**.21****	**.37****	,07	-,03	**-.22****	**-.13***	-,12	,10	**-.40****	**-.20****
DIF		1	**.16***	-,05	,08	,05	-,08	**.43****	,01	-,07	**.33****	-,01	,03	-,09	**-.16***	**.35****	**-.43****	**.15***
DDF			1	,09	**.32****	**.49****	-,01	**.13***	**.29****	**.19****	-,13	-,05	-,06	**-.14***	-,05	**-.13***	**-.39****	-,12
EOT				1	**.39****	**.22****	**.16***	-,05	**.15***	**.55****	**-.14***	-,01	**-.35****	-,04	,00	-,10	,01	**-.14***
BVAQ					1	**.57****	**.59****	**.47****	**.46****	**.73****	**-.21****	**-.29****	**-.52****	**-.24****	,12	**-.19****	**-.44****	**-.63****
Verbalizing						1	,01	**.29****	**.23****	**.36****	-,03	**-.15***	**-.19****	**-.19****	,05	-,01	**-.52****	**-.29****
Fantasizing							1	-,01	,09	**.27****	**-.25****	**-.16***	**-.49****	-,08	**.18****	**-.26****	,01	**-.33****
Identifying								1	,11	,11	**.24****	**-.23****	-,08	-,06	**-.16***	**.20****	**-.56****	-,08
Emotionalizing									1	**.23****	**-.20****	**-.28****	-,10	**-.26****	,02	**-.17****	**-.18****	**-.17****
Analyzing									.	1	**-.29****	-,09	**-.46****	**-.19****	**.17***	**-.21****	**-.19****	**-.78****
Neuroticism											1	**-.25****	,09	,02	**-.36****	**.67****	**-.15***	**.33****
Extraversion												1	**.17***	**.18****	,02	**-.16***	**.16***	,12
Openness													1	,08	**-.16***	,10	**.16***	**.40****
Agreeableness														1	,01	-,12	**.13***	**.15***
Conscientiousness															1	**-.31****	-,01	**-.15***
PST																1	**-.16***	**.25****
Clarity of feelings																	1	,10
Attention to feelings																		1

Note. TAS-20 = Toronto Alexithymia Scale; BVAQ = Bermond-Vorst Alexithymia Questionnaire; Neuroticism, Extraversion, Openness, Agreeableness and Conscientiousness are facets of NEO-Five Factor Inventory (NEO-FFI), PST = Positive Symptoms Total of Symptom Check List-90-Revised (SCL-90-R). Significance levels: ** = p< .01, *= p<.05.
